# Supplementary material for: Blocking HSV-1 glycoprotein K binding to signal peptide peptidase reduces virus infectivity in vitro and in vivo
Source: PLoS Pathog. 2021 Aug 5;17(8):e1009848. doi: 10.1371/journal.ppat.1009848 (PMC8370620; doi:10.1371/journal.ppat.1009848)
Supplement: Attachment — Submitted filename: Response (05-24-2021)PP_Final.doc [file ppat.1009848.s001.doc]

**RE**: PPATHOGENS-D-21-00605 (**Blocking HSV-1 glycoprotein K binding to signal peptide peptidase reduces virus infectivity *in vitro* and *in vivo***)

Dear Drs. Damania, Haldar and Malim:

Thank you very much to consider our appeal and below are our detailed responses to each of the reviewers' comments, indicating the changes we have made to the manuscript. Thank you for the opportunity to revise and resubmit our manuscript.

**REVIEWER #1:**

Summary: The Ghiasi laboratory has reported previously in a number of publications on HSV-1 glycoprotein K (gK) vis-a-vis its role in the enhancement of virus-induced corneal disease. The mechanism of action of this phenomenon involves the binding of gK to signal peptide peptidase (SPP). In the present investigation, the authors extended these studies to determine if blocking gK binding to SPP would impact virus infectivity and pathogenicity of corneal disease development in a therapeutic fashion. A series of experiments whose purpose was to fine map the gK binding site to SPP succeeded in identifying a 15 amino acid sequence within the gK molecule that binds to SPP that was designated dTat-gK4 (the 4th 15-mer peptide out of 33 15-mer peptides examined for binding to SPP). Importantly, dTat-gK4 reduced the infectivity of three different strains of HSV-1 that included the virulent McKrae strain of virus. This initial finding allowed for the performance of a subsequent series of experiments involving both BALB/c and C57BL/6 mice that demonstrated the potential therapeutic use of dTat-gK4 when administered to the ocular surface as eye drops. dTat-gK4-treated animals when compared with untreated control animals showed reduced corneal virus replication, increased survival, decreased eye disease (corneal scarring), reduced latency within trigeminal ganglia and a reduction in exhaustion markers of latently infected ganglia. The authors conclude that blocking gK binding to SPP using a gK peptide in the clinical setting could represent a new and effective therapeutic approach to manage HSV-1 ocular disease as well as other HSV-1-associated infections.

Review: This is a well written manuscript that nicely summarizes a large body of impressive and comprehensive investigations that provides new and exciting evidence for use of a gK peptide to manage HSV-1 ocular disease. The multiple experimental approaches lead to convincing data that support the overall premise of the investigation regarding the possible clinical therapeutic value of a gK peptide. While already a strong investigation, attention to some concerns might serve to further strengthen the manuscript for readers.

**Concern 1**. The McKrae strain of HSV-1 has been used by many laboratories to investigate the pathogenesis of corneal HSV-1 disease and most investigators therefore are familiar with its use in this area of investigation. The present study uses two additional viruses, however, that are designated HSV-1 GFP and HSV-1 VC1. On what virus strain background was HSV-1 GFP constructed? Some additional information on HSV-1 VC1 would also be helpful including a reference of its origin.

*RESPONSE*: Agree. A more detailed description of HSV-GFP and VC1 are now included in the revised manuscript.

**Concern 2**. Because HSV-1 strains other than the McKrae strain require corneal scarification for their effective use in corneal disease investigations, the authors should emphasize in the Discussion section that corneal scarification is not used in the present investigation, an experimental approach that heightens the clinical significance of their work.

*RESPONSE*: Agree. This is now mentioned in the discussion. Please see lines 369-371.

**Concern 3**. Although data showing the fine mapping of the gK binding region to SPP are convincing, to what extent does the secondary structure of gK also contribute to efficient binding of these two molecules?

*RESPONSE*: This is an interesting point but based on our published study on different forms of gK this is unlikely. This is now mentioned in the revised manuscript. Please see lines 108-109.

**Concern 4**. What is the rationale for measuring blepharitis at 4 days after ocular infection?

*RESPONSE*: In our hands peak of blepharitis is on day 4. This is now mentioned in the revised manuscript. Please see lines 496-497.

**Concern 5**. In vivo studies use an experimental plan whereby dTAT-gK4 is applied to the cornea prior to HSV-1 infection and thereafter at days 1, 2, 3, 4, and 5. To what extent is this experimental protocol clinically relevant? The findings provide impressive proof-of-principle, but patients with HSV-1 corneal disease would not be treated with this protocol in the clinical setting. The authors might comment how their very important findings might translate to treatment of patients in the clinic.

*RESPONSE*: The following sentences was added to the discussion section…. In this study we examined the prophylactic potential of blocking gK-SPP interaction, but since most adults are seropositive to HSV-1 infection, further studies will be needed to test the therapeutic potential of blocking this interaction. However, using gK4 as an eye drop outside of a clinic setting will be easy to do. Please see lines 394-398.

**Concern 6**. The first line of the Abstract states "HSV glycoprotein K (gK) is an essential herpes gene", a statement that is not correct. The glycoprotein is the product of a gene but not the gene itself. This should be corrected.

*RESPONSE*: Agree. “Gene” was changed to “protein”. Please see line 19.

**REVIEWER #2:**

Wang et. al. present work building on several previously published papers regarding the role of HSV-1 gK in the pathogenesis of viral keratitis. In this work, they extend the finding that gK binds to signal peptidase and use a peptide based strategy as a potential therapeutic for the disease. The major finding presented is that treatment with the peptide reduces infection in cell culture and in animal models reduces viral titers, clinical disease severity, and cytokines in the cornea. The work is potentially interesting but there are significant issues with the study design and other matters.

**Issue 1**. The most significant weakness in the study design is the lack of a proper control inactive peptide. In order to deliver their gK peptide, they coupled it to the TAT peptide that is known to facilitate transport of materials into cells (not properly cited). They then use the TAT peptide only as their negative control. This is not appropriate because they have an altered gK peptide that is inactive and this peptide coupled to TAT is the appropriate control.

*RESPONSE*: TAT peptide, not a scrambled peptide, is routinely used as a negative control in published studies against both RNA and DNA viruses . None of these studies used scramble peptide instead of TAT as control. These studies are now cited in the revised manuscript. Please see lines 352-358.

**Issue 2**. A second issue is the lack of proper referencing. There is a considerable literature on the use of peptides as antivirals and more specifically, as potential treatments for viral keratitis. None of these papers are cited. The authors completely ignored this body of literature and do not place their results in the context of the existing literature.

*RESPONSE*: We have added proper citations into the manuscript text regarding use of TAT to facilitate transport of materials into cells. The following sentences were added to the discussion section….”In addition to the use of acyclovir and ganciclovir, nanocarriers, prodrugs, peptide conjugation, and in situ gelling systems have been used to slow the onset or progression of HSK and HSV-1 replication *in vitro* and in animal models . Therefore, these studies and many more have evaluated the antiviral activities of natural or synthesized compounds as potential treatments for viral keratitis. However, to our knowledge this is the first study showing that inhibition of SPP-gK interaction with a synthetic gK peptide has antiviral activity”. Please see lines 389-393.

**Issue 3**. A third issue is their data indicate the TAT peptide is inactive when other publications have shown it, and some derivatives of TAT, have antiviral activity. This discrepancy is not explained or even addressed.

*RESPONSE*: In our search we observed many studies showing that TAT alone does not have any antiviral activity and we have now added some of these references to our discussion and read as follow….”In this study we have used TAT peptide as a control and we did not observe any antiviral activity associated with using three different strains of HSV-1, three different cell lines and two different strains of mice. Similar to this study, TAT alone did not have any antiviral activity against two different strains of HSV-1, adenovirus type 5, cytomegalovirus, vaccinia virus, and simian virus 40 in cell culture models . In addition, no antiviral activities associated with the TAT control peptide was reported for influenza virus , Ebola virus , Epstein-Barr virus , or Japanese encephalitis virus .” Please see lines 352-358.

**Issue 4**. There is no vehicle only control in the animal experiments and they do not provide the gender of the mice. They should have tested both male and female mice or explained why this is not necessary.

*RESPONSE*: The gender of mice was indicated in M&M section and under the heading of “Cells, virus, and mice”. We indicated that we used female mice. We used female mice since in numerous studies that we have done we did not see any significant differences between male and female mice and since female mice are easier to house and handle, we used female mice. The similarity between male and female mice is now indicated in the M&M section. Please see lines 413-415.

**Issue 5**. It is absolutely essential that all peptide concentrations should be given in micromolar not micrograms/volume. Using the weight makes it difficult to compare the efficacy of their peptide with other antivirals or antiviral peptides. They do not discuss the efficacy of their peptide in the context of the efficacy of other antiviral peptides.

*RESPONSE*: The following sentence was added to the M&M section and reads as follow….”Ten µg/ml of dTat-gK4 peptide is approximately 3.1 µM, while 10 µg/ml of dTat peptide is approximately 6.4 µM”. Please see lines 443-444.

**Issue 6**. The presentation of the data in Figure 3 is potentially misleading to readers. At 50 micrograms per ml. concentration the reduction is less than 1 log. By plotting the Y axis to extend only over a 1 log span makes the differences look greater than they are so unless readers pay attention to the scale they will think the peptide is working well when in fact it is similar to other antiviral peptides in the literature. Again, all concentrations should be micromolar.
*RESPONSE*: Disagree. We did not claim that this peptide is working better than other peptides in the literature. The only way that we could claim this would be to compare our peptide with other peptides but this was not the goal of this study. In addition, most antiviral peptides in the literature are not based on blocking a specific host gene with a specific viral gene as we have done here. Basically, this reviewer wants us to compare apples to oranges.

**Issue 7**. They do not provide an EC50 value as is commonly done with antivirals.

*RESPONSE*: The following sentence was added to the legend to Figure 3 and reads as follow….“Half maximal effective concentration (EC 50) of dTat-gK4 peptide was approximately 2.1 µM (6.6 µg/ml)”. Please see lines 844-845.

**Issue 8**. In the second sentence of the introduction the authors make the statement "...Although the HSV-1 genes involved in eye disease is not yet known...." This statement is absolutely incorrect. There are several papers and review articles describing the role of HSV genes in ocular infections. Once again, the existing literature is not properly cited.

*RESPONSE*: Disagree. To the best of our knowledge there is no known herpes gene that is universally accepted to cause eye disease. For example, deletion of any of the essential herpes genes will affect virus replication in the eye, indirectly resulting in less or no eye disease but this does not mean that the deleted gene is involved in eye disease. McKrae virus is known to cause eye disease, while KOS and RE do not cause eye disease. All three viruses have been sequenced by different groups but nobody was able to correlate any specific region of McKrae to eye disease. Although some studies claim to have mapped the eye disease gene, there is no concrete data showing any specific gene or region of virus directly involved in eye disease.

Thank you very much for allowing us to revise and resubmit our manuscript. We trust that we have satisfactorily interpreted these helpful reviewers’ comments, and we have now made all the changes that were requested by reviewer #1 and also most of the changes that was requested by reviewer #2. New text in the body of the manuscript is printed in “Blue Arial” rather than “Black Arial”. We hope that the manuscript will now be found to be acceptable for publication in PLOS Pathogen.

Best Regards,

Homayon Ghiasi
