# Supplementary material for: Blocking HSV-1 glycoprotein K binding to signal peptide peptidase reduces virus infectivity in vitro and in vivo
Source: PLoS Pathog. 2021 Aug 5;17(8):e1009848. doi: 10.1371/journal.ppat.1009848 (PMC8370620; doi:10.1371/journal.ppat.1009848)
Supplement: Attachment — Submitted filename: Response (07-22-2021)2nd Rev.doc [file ppat.1009848.s002.doc]

**RE**: PPATHOGENS-D-21-00605R1 (**Blocking HSV-1 glycoprotein K binding to signal peptide peptidase reduces virus infectivity *in vitro* and *in vivo***)

Dear Drs. Lin, Damania, Haldar and Malim:

Thank you very much for giving us the to revise and submit our manuscript. Below are our detailed responses to each of the reviewers' comments, indicating the changes we have made to the manuscript.

**REVIEWER #1:**

This is a revised manuscript that provides new and important information on the use of a gK peptide to manage HSV-1 corneal disease, findings that may have significant clinical relevance. The manuscript is well written, and the results and conclusions are convincing and compelling. All concerns and questions raised by the previous reviewers have been answered thoughtfully and thoroughly with appropriate revisions throughout the manuscript. No additional weaknesses or concerns have been identified.

**This reviewer had no concern regarding the revised manuscript.**

**REVIEWER #2:**

Wang et. al. present an intriguing study on the use of a novel peptide to decrease HSV-1 disease in a murine model of HSV-1 eye disease. The authors build on their previous extensive work on gK and its binding to SPP to map the region of gK responsible for SPP binding. They then synthesize a novel molecule that contains the gK 15mer (gK4) responsible for binding to SPP bound to HIV TAT to improve uptake in cells. They then go on to demonstrate that this molecule inhibits HSV-1 replication in vitro and in vivo and improves clinical HSV disease scores. Finally, they present some gene expression data to suggest the mechanism of disease prevention does not impact the host immune response to the virus. While there are doubts about the feasibility of using this specific compound in a clinical setting, the mapping of the interaction between gK and SPP and this proof of principle study are highly relevant to the HSV-1 field and highlights a novel mechanism of pathogenesis. Overall, this was a well-designed and executed study, and the manuscript is easy to follow.

I did not identify any serious methodological flaws. No obvious controls are lacking (save for one discussed below). For the most part their conclusions are supported by their data apart from a few exceptions below. I do have some suggestions to improve the presentation of the data and ensure proper statistical analysis which are discussed below. However, I do not believe they will significantly alter the results or conclusions.

**Minor concern 1**. Abstract line 23: The use of “fine mapped” is non-specific and the authors have a definitive length of a 15mer so no reason to be non-specific. Recommend removing the modifier “fine.”

*RESPONSE*: Agree. The word “fine” is now deleted.

**Minor concern 2**. Intro line 45. I agree with reviewer #2’s comment that to say that the HSV genes are not known seems to discount a large body of literature. Perhaps something like “While the precise viral pathogenicity determinant in ocular disease remains to be elucidated…”

*RESPONSE*: Agree. The above sentence was added as follow…. “While the precise viral pathogenicity determinant in ocular disease remains to be elucidated,”.

**Minor concern 3**. Intro line 65: This sentence starts with “We also asked…” but then there is no question. Perhaps the authors mean “We also sought to determine?” There is a similar issue in the results on line 169.

*RESPONSE*: Agree to both and were changed to “We also sought to determine” in line 65 and to “We sought to determine” in line 169.

**Minor concern 4**. Intro line 72: The sentence that reads “Thus, we have identified an effective therapeutic target to reduce HSV-1 primary infection, latency, and HSV-1-induced eye disease .” is problematic. First, it feels like it should go into the discussion. Second, it is not supported by the data in the manuscript. This study is entirely done using a mouse model that, while is good as far as mouse models go, does not perfectly translate to human infection. No primary human corneal cells are used here nor other human tissues. Not to mention that using eye drops at the time of infection is entirely not feasible in humans. This sentence needs to be toned down. The sentence immediately following is entirely appropriate.

*RESPONSE*: Agree. This sentence is now deleted.

**Minor concern 5**. Figure 3: Why did the authors stop at 50ug/mL? Would increasing the dose further reduce infectivity? Some type of viability control should be provided. Is this compound toxic and thus killing the cells? Why did the authors leave the viral inoculum on for the whole time? I think that a 2 hour incubation would be sufficient. Where are the error bars or individual data points in this figure? Are there stats done here?

*RESPONSE*: We did not leave the viral inoculum plus peptide on for the whole time. It was done for one hour followed by adding fresh media plus peptide. We did not mention this in our original manuscript but now we modified in the revised manuscript and mentioned it in the legends to Figs. 3-5.

The peptide at 100ug/mL showed toxicity and that was the reason that we did not use more than 50ug/ml of dTAt-gK4. This is now mentioned in the revised manuscript under M&M section.

We looked at cells viability-death under the microscope but did not measure calculate the ratio of cell survival to cell death and the following sentence was added to the result section (Lines 140-142)… “In contrast to no side effect associated with cell viability at 50 µg/ml concentration of dTat-gK4 peptide, dTat-gK4 peptide at concentration of 100 µg/ml had toxicity on treated RS, 293 and Vero cells (not shown)”. This figure was revised and error bars were added.

**Minor concern 6**. Figure 4: The use of error bars (SEM or Sd) is less than ideal. Would be better to show individual data points as is done in Figure 6B.

*RESPONSE*: Agree. This is now presented as data points similar to Fig. 6B.

**Minor concern 7**. Figure 5: These data need to be quantified. The authors make the statement “more cells were positive than in cells incubated with dTat-gK4,” but the reader is left with only 1 image. It sounds as though the authors have already quantified these data, but it should be displayed as such.

*RESPONSE*: Agree. The data were quantified and now presented as Fig. 5B and Fig. 5 is now Fig. 5A. These quantification is now described in the revised text (Lines 168-172).

**Minor concern 8**. Figure 6B: The statistical test done here is a Student’s t-test according to the methods, but these data do not look normally distributed and there are not more than 30 samples per group. Therefore, either some test of normality should be performed, or a non-parametric test should be performed.

*RESPONSE*: A non-parametric test (Mann Whitney) was perform using Prism 9, and the P value is 0.028. This is now indicated in the revised manuscript

**Minor concern 9**. Figure 6C: This is truly a minor point. The way these data are displayed gives the impression that this is a Kaplan-Meyer survival curve, but the authors did not do a survival analysis. They performed a Fisher’s exact test at a specific time point (which I think is the correct test here). Alternatively, the data could simply be displayed in a table. The way it is currently displayed makes the reader think that the mice treated with control actually died FASTER…but again, that wasn’t the test that was done.

*RESPONSE*: We agree with the reviewer observation but actually the mice in control group dies faster than the treated group and this figure shows that.

**Minor concern 10**. Results line 197: The authors mention measuring mRNA for various HSV proteins (gK, gB, etc.) I believe since they are looking at gene expression they should use the gene designations (UL20 etc.)

*RESPONSE*: We agree with the reviewer that we are looking at RNA rather than protein but looking at the published studies everyone referring to UL53 as gK and UL27 as gB. In the M&M section we have mentioned gK as UL53 and gB as UL27.

**Minor concern 11**. Figure 8: The use of bar graphs with error bars is not very transparent and can hide obvious outliers. This would better be displayed like the data in figure 6B.

*RESPONSE*: Agree. Figure 8 is now presented as data points similar to Fig. 6B.

**Minor concern 12**. Discussion: There is too much restating of findings early on, see lines 315 and the rest of that paragraph.

*RESPONSE*: Agree. This paragraph is now deleted.

**Minor concern 13**. Discussion line 363: what is meant by “virus load?” Input virus? Titer of inoculation virus? DNA? PFU? Please clarify.

*RESPONSE*: Virus load was change to “input virus”.

**Minor concern 14**. Discussion line 380: the comment “these results suggest that T cell responses and viral clearance are enhanced” is not supported by the data. The authors did not directly measure T cell responses in this study. Gene expression profiling on bulk tissue does not isolate T cell responses. They did not measure whether T cells were more or less responsive to viral antigen. They also did not demonstrate that gK4 treatment enhanced viral clearance. My understanding is that the proposed mechanisms is that gK4 inhibits viral replication at the site of inoculation by interfering with gK binding to SPP. This has nothing to do with cells of the innate or adaptive immune system sensing and/or clearing the virus. This sentence needs to be removed.

*RESPONSE*: Agree. Done

Thank you very much for allowing us to revise and resubmit our manuscript. We trust that we have satisfactorily interpreted these helpful reviewers’ comments, and we have now made all the changes that were requested by requested by reviewer #2, while no changes were requested by reviewer #1. New text in the body of the manuscript is printed in “Blue Arial” rather than “Black Arial”. We hope that the manuscript will now be found to be acceptable for publication in PLOS Pathogen.

Best Regards,

Homayon Ghiasi
